# The incremental predictive value of biological aging indicators for cognitive impairment in older adults: a longitudinal analysis on the Mr. OS & Ms. OS cohort

**DOI:** 10.1186/s13195-025-01917-1

**Published:** 2025-12-06

**Authors:** Yafei Wu, Ting Zhang, Tung Wai Auyeung, Jenny Lee, Jason Leung, Timothy Kwok

**Affiliations:** 1 Department of Medicine and Therapeutics, The Chinese University of Hong Kong, Shatin, New Territories, Hong Kong SAR, China; 2 Department of Geriatrics, Renji Hospital, School of Medicine, Shanghai Jiaotong University, Shanghai, 200127 China; 3 Jockey Club Institute of Ageing, The Chinese University of Hong Kong, Shatin, New Territories, Hong Kong SAR, China; 4 Jockey Club Centre for Osteoporosis Care and Control, The Chinese University of Hong Kong, Shatin, New Territories, Hong Kong SAR, China

**Keywords:** Biological aging, Frailty index, Biochemical marker, Telomere length, Cognitive impairment

## Abstract

**Background:**

Biological aging (BA) markers such as frailty and telomere length are closely linked to cognitive impairment (CI). However, the incremental value of biochemical marker-enriched frailty index (FI) and telomere length for predicting CI remains underexplored. We aimed to assess the incremental value of BA markers beyond conventional cognitive tests for CI risk stratification.

**Methods:**

A total of 1674 community-dwelling older adults without baseline CI were obtained from the Mr. OS & Ms. OS (Hong Kong) cohort. Baseline BA measures included frailty phenotype, three FI versions (without/with 2 or 4 serum biochemical markers: creatinine, homocysteine, high-sensitivity C-reactive protein, and 25-hydroxyvitamin D), and leukocyte telomere length. CI was assessed by concurrently using the MMSE and CSI-D tests at the 7-year follow-up. Penalized logistic regression was used to evaluate the incremental value of BA indicators beyond cognitive tests for CI prediction, with the area under the precision-recall curve (AUPRC) as the primary performance metric.

**Results:**

The mean age of the study sample was 70.7 years (SD: 4.2), and 44.1% were females. The 7-year incidence of CI was 17.0% (285/1674). Compared to baseline CSI-D score, the FI incorporating four biochemical markers demonstrated significant incremental value for CI prediction (AUPRC improvement: 0.037, *P* < 0.001), while other frailty indicators (frailty phenotype and other two FI versions) showed no significant added value. Telomere length provided additional predictive value across all frailty-related models (AUPRC improvements: 0.009–0.078, all *P* < 0.001) except the FI without biochemical markers. The optimal prediction model, which considered the FI with four biochemical markers and telomere length, achieved an AUPRC (SD) of 0.568 ± 0.092 and an AUROC (SD) of 0.826 ± 0.037.

**Conclusions:**

Frailty index with four biochemical markers and telomere length showed moderate incremental value over cognitive tests for CI prediction in older adults. The findings highlight the importance of multisystem biological aging assessment in CI risk stratification. Further validation is warranted in large cohorts.

**Supplementary Information:**

The online version contains supplementary material available at https://doi.org/10.1186/s13195-025-01917-1.

## Introduction

Chronological age, though a straightforward measure of time elapsed since birth, fails to fully capture the heterogeneity in individual aging process. In contrast, biological age reflects the functional state of an organism, integrating clinical, physiological, and molecular biomarkers to quantify the cumulative burden of aging-related changes [1]. Emerging evidence suggests that biological age, as opposed to chronological age, is a more accurate predictor of age-related disease risk [2], mortality [3], and functional decline [4].

Despite growing interest in quantifying biological aging (BA), no consensus exists on a gold-standard biomarker or composite measure. Multiple indices have been proposed, each reflecting distinct aspects of aging physiology. Among them, the frailty phenotype operationalizes biological age through physical decline (e.g., weight loss, exhaustion) [5], while the Rockwood frailty index (FI) quantifies accumulated health deficits across clinical and functional domains [6]. Notably, these clinical measures primarily capture macroscopic manifestations of aging, whereas molecular-level changes may precede observable functional deterioration by years. Telomere length, a molecular marker of cellular senescence, has also been widely used as a proxy for biological aging [7], though its relationship with premature aging is still debated [8].

The relationship between biological aging and cognitive impairment (CI) has been increasingly recognized. Although individual BA marker such as frailty phenotypes, FI scores, or telomere length has been independently linked to CI [9–11], existing studies exhibit notable limitations. Firstly, studies employing the FI have predominantly relied on self-reported clinical deficits [12], overlooking the potential synergy between clinical frailty and underlying biochemical alterations in aging such as chronic inflammation (e.g., hs-CRP) [13], metabolic dysregulation (e.g., homocysteine) [14], or nutritional insufficiency (e.g., 25(OH)-D) [15]. This oversight is critical, as a biochemical marker-enriched FI might capture aging-related pathophysiology more comprehensively than conventional clinical deficit measures. While no study to date has examined whether integrating these biochemical markers into FI constructs enhances the prediction power for CI. Besides, few studies have systematically compared the predictive value of multiple BA indicators for CI risk stratification within the same cohort. This lack of concurrent comparison makes it difficult to objectively evaluate which BA metrics are the most robust predictors for CI on a unified scale.

Therefore, in this study, we aimed to assess the incremental predictive value of various BA indicators beyond conventional cognitive tests for CI risk stratification in 1674 individuals representing the Hong Kong Chinese community-dwelling older adults who do not have CI at baseline. Our study aimed to address these gaps by: (1) constructing a FI that integrates both clinical deficits and aging-related biochemical markers, and then comparing its discriminative capacity against the FI without biochemical components for CI prediction, and (2) comprehensively evaluating the prediction values of multiple BA indicators (frailty phenotype, different FI versions, and telomere length) in predicting CI.

## Methods

### Study design and participants

Data used in this study was obtained from the Mr. OS & Ms. OS (Hong Kong) cohort, an ongoing community-based prospective study designed to investigate the determinants of osteoporosis and related health outcomes in aging populations. Between August 2001 and March 2003, the study enrolled 4000 community-dwelling older adults (2000 men and 2000 women) aged ≥ 65 years. A stratified sampling approach was employed to ensure equal representation across three age groups: 65–69, 70–74, and ≥ 75 years. The study was carried out in accordance with the Declaration of Helsinki and was approved by the Clinical Research Ethics Committee of The Chinese University of Hong Kong. All participants provided informed consent. More detailed description of the cohort was shown in previous publications [16].

**Fig. 1:**
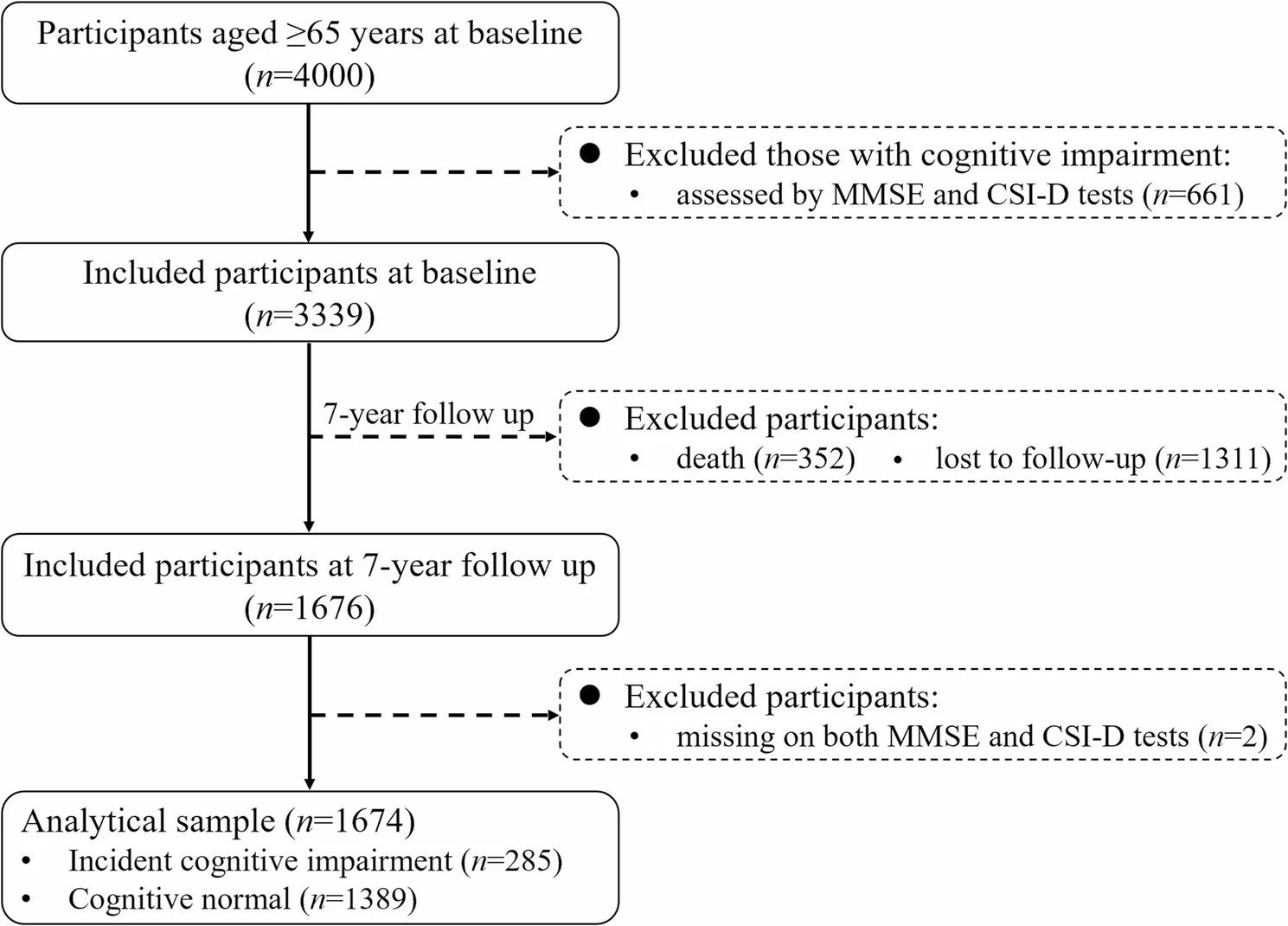
The workflow of study sample selection

In this study, we first excluded 661 participants with baseline CI. Subsequently, we excluded an additional 1665 participants who did not complete the 7-year follow-up for the following reasons: 352 had died, 1311 were lost to follow-up, and 2 lacked cognitive test data. The final analytical sample comprised 1674 participants (Fig. 1). Table S1 presents the comparison of baseline characteristics between included and excluded participants, quantified by various effect size measures [17]. With the exception of age, all baseline characteristics demonstrated small to small-medium effect sizes between the included and excluded participants.

## BA indicators

In this analysis, we evaluated multiple BA indicators at baseline, including Fried frailty phenotype, three FI versions (without/with 2 or 4 serum biochemical markers), and leukocyte telomere length. Fried frailty phenotype was assessed using the 5-item Cardiovascular Health Study (CHS) criteria, characterized by five components: unintentional weight loss, exhaustion, low physical activity, slow walking speed, and weak grip strength [5]. Following previous studies, we defined the equivalent measures in our study as follows: body mass index (BMI) < 18.5 kg/m², self-reported lack of energy, and the lowest quartiles of the Physical Activity Scale for the Elderly (PASE) score, walking speed, and grip strength [18]. Participants were further categorized as frail (≥ 3 criteria), pre-frail (1–2 criteria), or robust (0 criteria). The FI was constructed according to the Rockwood’s Cumulative Deficit Theory [19]. We first established a FI without biochemical markers using 35 clinical measures (abbreviated as “FI_no_bio”). Then, we created two additional FI versions by progressively incorporating four aging-associated biochemical markers (creatinine, homocysteine, hsCRP, and 25-hydroxyvitamin D [25(OH)-D]) [13–15, 20]. Specifically, we first incorporated two biochemical markers (creatinine and homocysteine, abbreviated as “FI_with_2_bio”) into the 35-itme-based FI due to their low missingness (< 8%). Subsequently, a more comprehensive FI (“FI_with_4_bio”) was created by further adding hsCRP and 25(OH)-D (~ 30% missing). When constructing FI, all continuous variables were dichotomized using clinically established or validated cutoff values. More detailed information on all included variables and the development process of three FI versions is presented in Table S2 and Text S1. Telomeres are repetitive DNA sequences at chromosome ends, and shortened telomere length is considered as an aging marker [21]. In this cohort, telomere length was measured using a molecular inversion probe-quantitative PCR assay (MIP-qPCR) on DNA extracted from peripheral blood leukocytes, with results expressed as absolute length in kilobases (kb) [22].

### Assessment of cognitive function

Cognitive impairment (CI) was defined by concurrent assessments using two validated instruments: the Hong Kong version of Mini-Mental State Examination (MMSE) and the Community Screening Instrument for Dementia (CSI-D). The Hong Kong version of the MMSE was culturally adapted for Cantonese-speaking populations, while preserving the standard 30-point scale. This validated instrument, with education-adjusted cutoff scores, demonstrated excellent discriminant validity (AUC = 0.91) for dementia [23]. The CSI-D scale, developed by the 10/66 Dementia Research Group, consists of a 32-item cognitive test administered to the participant and a 26-item informant interview, assessing the participant’s daily functioning and general health [24]. The CSI-D yields three summary scores: (1) the cognitive score (cogscore), a composite score derived from participant testing with differential item weighting, (2) the informant score (relscore), an unweighted total score based on informant answers, and (3) the discriminant function score (dfscore), calculated as 0.452322-(0.01669918×cogscore)+(0.03033851×relscore). The combination of cognitive and informant scores in a discriminant score produces better sensitivity and specificity than cognitive score alone. When relscore is not available, cogscore alone serves as the alternative measure. Participants were classified as CI if they met any following criteria: (1) MMSE ≤ 18 for illiterate subjects, or ≤ 20 for those with 1–2 years of schooling, or ≤ 22 for those with more than 2 years of schooling [23], (2) dfscore ≥ 0.120 or cogscore ≤ 29.5 [25]. This dual-assessment approach enhanced the sensitivity of CI identification by capturing both general cognitive decline (MMSE) and dementia-specific impairment (CSI-D), thereby reducing false negatives in population screening.

### Statistical analysis

All analyses were conducted with R and Python languages. Mean (standard deviation) and number (percentage) were used to summarize the continuous and categorical variables. Baseline characteristics across cognitive status (normal vs. incident cognitive impairment) were compared using t-test, Kruskal-Wallis test, or chi-square test. Two-sided value of *P* < 0.05 was regarded as statistically significant. Frailty index was plotted using histogram and described by validated cut-off values (fit: <0.1, pre-frail: 0.1–0.2, and frail: ≥0.2) [26].

We employed penalized logistic regression (incorporating L_1_ or L_2_ penalty) to examine the incremental predictive value of BA indicators in estimating incident CI risk. Unlike conventional statistical methods, penalized logistic regression, a machine learning algorithm, is designed to automatically retain important predictors via L_1_’s sparsity-inducing property (Lasso) or mitigate potential multicollinearity effects via L_2_’s coefficient shrinkage (Ridge), thereby reducing overfitting risks [27]. We developed sequential CI prediction models, starting with a reference model (Model 1) that included age, sex, education, and the baseline cognitive score, a set of well-established predictors in CI prediction studies [28–30]. Subsequently, we created Models 2–5 by individually incorporating each of the four frailty-related BA indicators (frailty phenotype, FI without biomarkers, FI with two biomarkers, and FI with four biomarkers) into Model 1. Models 6–9 were developed by further adding telomere length to Models 2–5, respectively. In addition, the discriminatory power of each BA indicator and baseline cognitive test score was assessed individually.

We split the data into training and test sets in a ratio of 7:3 using stratified random sampling. Key hyperparameters (class weight, regularization strength, and penalty term) of penalized logistic regression were optimized via a 5-fold cross-validated grid search on the training set. All continuous predictors were normalized using MinMaxScaler technique (scaled to a range of 0 to 1) prior to being entered into the penalized logistic regression model. The scaler model was fitted exclusively on the training data and subsequently applied to the test set to prevent data leakage. Given the relatively low incidence of CI (approximately 17%), which introduces class imbalance, we prioritized the precision-recall (PR) curve and its area under the curve (AUPRC) as primary evaluation metric [31], which provides more objective assessment by considering both majority and minority classes in imbalanced prediction. Other metrics such as balanced accuracy (BACC), sensitivity (SEN), positive predictive value (PPV), F1-score, and the receiver operating characteristic (ROC) curve and its area under the curve (AUROC) were also calculated. All metrics were obtained through a 1000-time bootstrapping to ensure stable estimates, with final results presented as means ± standard deviations. The differences of AUPRCs between models were compared on test set, with effect sizes quantified using Cohen’s *d* and categorized as small (*d* = 0.2), medium (*d* = 0.5), or large (*d* ≥ 0.8) [32].

## Results

### Distribution of three frailty index versions

**Fig. 2:**
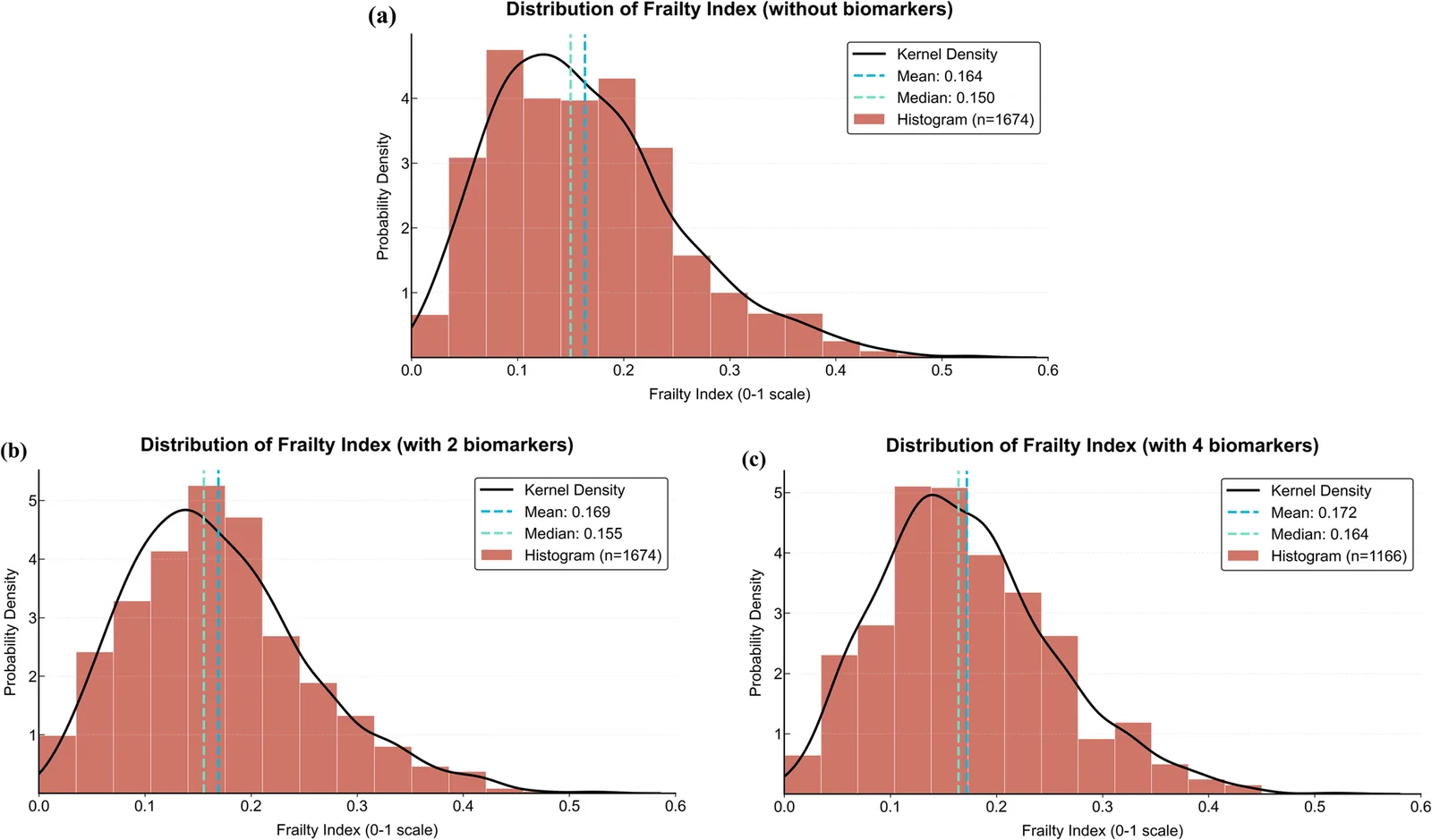
Distribution of frailty index. **a** FI without biochemical markers, **b** FI with two biochemical markers (eGFR and homocysteine), and **c** FI with four biochemical markers (eGFR, homocysteine, hsCRP, and 25(OH)-D)

The distribution of three FI versions (without/with two or four serum biochemical markers) was shown in Fig. 2, with mean FI scores of 0.164 (SD: 0.088), 0.169 (SD: 0.085), and 0.172 (SD: 0.082), respectively. The proportion of fit, pre-frail, and frail were 25.7%, 43.0%, and 31.2% (without biochemical markers), 22.6%, 44.7%, and 32.7% (with two biochemical markers), 18.1%, 50.1%, and 31.8% (with four biochemical markers), respectively.

### Descriptive characteristics of study sample

As shown in Table 1, the study included 1674 participants without baseline CI (mean age: 70.7 ± 4.2 years, 44.1% female). After a 7-year follow up, 285 of them (17.0%) had incident CI. Compared to the cognitively normal, the CI participants were much older, more likely to be female, and less educated (*P* < 0.001). They also had significantly lower baseline MMSE and CSI-D scores (*P* < 0.001). For BA indicators, all except telomere length showed significant differences between the two groups (*P* < 0.01), with CI people demonstrating much higher levels of frailty. Among the four biochemical markers, creatinine concentrations differed significantly between the two groups (*P* < 0.001).

**Table 1:** Baseline characteristics of study sample

Variables	Overall (*n* = 1674)	Normal (*n* = 1389)	Incident CI (*n* = 285)	*P*-value
Age (year, mean ± std)	70.7 ± 4.2	70.4 ± 4.0	72.3 ± 4.8	< 0.001
Female (*n*, %)	738 (44.1%)	564 (40.6%)	174 (61.1%)	< 0.001
Education (*n*, %)				< 0.001
Illiterate	244 (14.6%)	140 (10.1%)	104 (36.5%)	
Primary or below	811 (48.4%)	666 (47.9%)	145 (50.9%)	
Secondary or above	619 (37.0%)	583 (42.0%)	36 (12.6%)	
MMSE score (mean ± std)	27.1 ± 2.5	27.4 ± 2.2	25.4 ± 2.9	< 0.001
CSI-D score (mean ± std)	30.9 ± 1.3	31.1 ± 1.1	30.0 ± 1.5	< 0.001
Fried phenotype (*n*, %)				0.002
Fit	1093 (65.3%)	922 (66.4%)	171 (60.0%)	
Pre-frail	550 (32.9%)	448 (32.3%)	102 (35.8%)	
Frail	31 (1.9%)	19 (1.4%)	12 (4.2%)	
FI_no_bio (mean ± std)	0.164 ± 0.088	0.158 ± 0.084	0.191 ± 0.101	< 0.001
FI_with_2_bio (mean ± std)	0.169 ± 0.085	0.164 ± 0.082	0.194 ± 0.098	< 0.001
FI_with_4_bio (mean ± std)	0.172 ± 0.082	0.167 ± 0.078	0.197 ± 0.095	< 0.001
Creatinine (µmol/L, mean ± std)	72.86 ± 19.16	73.73 ± 19.24	68.58 ± 18.24	< 0.001
Homocysteine (µmol/L, mean ± std)	13.78 ± 4.20	13.75 ± 3.87	13.97 ± 5.56	0.539
hsCRP (mg/L, median, IQR)	1.50 (0.70–3.30)	1.50 (0.70–3.30)	1.50 (0.70–3.30)	0.521
25(OH)-D (nmol/L, mean ± std)	61.29 ± 14.03	61.29 ± 14.15	61.32 ± 13.54	0.974
Telomere length (mean ± std)	9.11 ± 1.88	9.06 ± 1.83	9.33 ± 2.06	0.118

The analytical sample sizes for “FI_with_4_bio”, “Telomere length”, “Creatinine”, “Homocysteine”, “hsCRP”, and “25(OH)-D were 1166, 826, 1552, 1552, 1163, 1165, respectively. Continuous and categorical variables were compared by t-test or Kruskal-Wallis test, and chi-square test

*CI* Cognitive impairment, *MMSE* Mini-mental state examination, *CSI-D* Community Screening Instrument for Dementia, *FI_no_bio* Frailty index without biochemical markers, *FI_with_2_bio* Frailty index with two biomarkers (eGFR and homocysteine), *FI_with_4_bio* Frailty index with four biomarkers (eGFR, homocysteine, hsCRP, and 25(OH)-D), *hsCRP* hypersensitive C-reactive protein, *25(OH)-D* 25-hydroxyvitamin D

### Incremental value of BA indicators in predicting CI

As shown in Table S3, baseline characteristics were well-balanced between training and test sets (all *P* > 0.05). The prediction performance of individual BA indicators and baseline cognitive test scores is shown in Table S4, and comparisons of AUPRCs between models and the corresponding optimal hyperparameters are shown in Table S5 and Table S6, respectively. We observed that the baseline CSI-D score demonstrated significantly stronger predictive performance than the MMSE score (AUPRC: 0.380 ± 0.049 vs. 0.332 ± 0.049, *P* < 0.001). In addition, all BA indicators (AUPRC range: 0.169–0.304) showed relatively lower predictive ability compared to the baseline CSI-D score, among which the FI incorporating four biochemical markers achieved the highest AUPRC (0.304 ± 0.054). Therefore, we further evaluated whether BA indicators provided incremental predictive value over the reference model that included age, sex, education, and baseline CSI-D score, with results presented in Table 2 and Tables S7-S9. Our analysis revealed that neither the frailty phenotype, the FI without biochemical marker, nor the FI with two biochemical markers improved CI prediction compared to the reference model. Only the FI with four biochemical markers (Model 5) yielded a statistically significant predictive gain (Model 5 vs. Model 1: Δ_AUPRC_ = 0.037, 95%CI: 0.032–0.042, *P* < 0.001). Further inclusion of telomere length across Models 2–5 (Models 6–9) demonstrated its consistent incremental predictive value, with AUPRC improvements ranging from 0.009 to 0.078 (all *P* < 0.001). The addition of the four-biomarker FI to the reference model (Model 5 vs. Model 1) yielded an effect size of 0.451 in AUPRC change, indicating a small-to-medium effect. When telomere length was further added to the frailty-related models, the effect sizes varied: it was 0.100 (small) for the frailty phenotype model (Model 6 vs. Model 2), 0.112 (small) for the two-biomarker FI model (Model 8 vs. Model 4), and 0.706 (medium-to-high) for the four-biomarker FI model (Model 9 vs. Model 5).

**Table 2:** Prediction performance of penalized logistic regression by progressively including BA indicators on test set

Model	AUPRC	AUROC	BACC	SEN	PPV	F1
M1: Ref	0.453 ± 0.055	0.782 ± 0.026	0.676 ± 0.029	0.563 ± 0.054	0.355 ± 0.040	0.435 ± 0.041
M2: Ref + Phenotype	0.439 ± 0.054	0.783 ± 0.025	0.668 ± 0.029	0.491 ± 0.055	0.394 ± 0.046	0.436 ± 0.045
M3: Ref + FI_no_bio	0.449 ± 0.054	0.784 ± 0.026	0.693 ± 0.028	0.655 ± 0.051	0.335 ± 0.035	0.443 ± 0.037
M4: Ref + FI_with_2_bio	0.445 ± 0.054	0.784 ± 0.025	0.678 ± 0.029	0.574 ± 0.055	0.352 ± 0.039	0.436 ± 0.041
M5: Ref + FI_with_4_bio	0.490 ± 0.062	0.755 ± 0.033	0.639 ± 0.033	0.431 ± 0.063	0.382 ± 0.059	0.404 ± 0.055
M6: Ref + Phenotype + Telomere	0.448 ± 0.076	0.810 ± 0.033	0.747 ± 0.032	0.862 ± 0.054	0.337 ± 0.044	0.483 ± 0.049
M7: Ref + FI_no_bio + Telomere	0.450 ± 0.076	0.812 ± 0.033	0.722 ± 0.038	0.703 ± 0.070	0.369 ± 0.053	0.482 ± 0.054
M8: Ref + FI_with_2_bio + Telomere	0.455 ± 0.076	0.813 ± 0.034	0.729 ± 0.037	0.748 ± 0.067	0.359 ± 0.049	0.483 ± 0.052
M9: Ref + FI_with_4_bio + Telomere	0.568 ± 0.092	0.826 ± 0.037	0.751 ± 0.040	0.786 ± 0.072	0.399 ± 0.061	0.527 ± 0.061

The results in the above table were expressed as mean and standard deviation by a 1000-time bootstrapping method

*BA* Biological aging, *Ref* Reference (the reference model included age, sex, education, and baseline CSI-D score), *AUPRC* Area under the precision-recall curve, *AUROC* Area under the receiver operating characteristic curve, *BACC* Balanced accuracy, *SEN* Sensitivity, *PPV* Positive predictive value, *F1* F1 score, *Phenotype* Fried phenotype, *FI_no_bio* Frailty index without biochemical markers, *FI_with_2_bio* Frailty index incorporating eGFR and homocysteine, *FI_with_4_bio* Frailty index incorporating eGFR, homocysteine, hsCRP and 25-OH Vitamin D

**Fig. 3:**
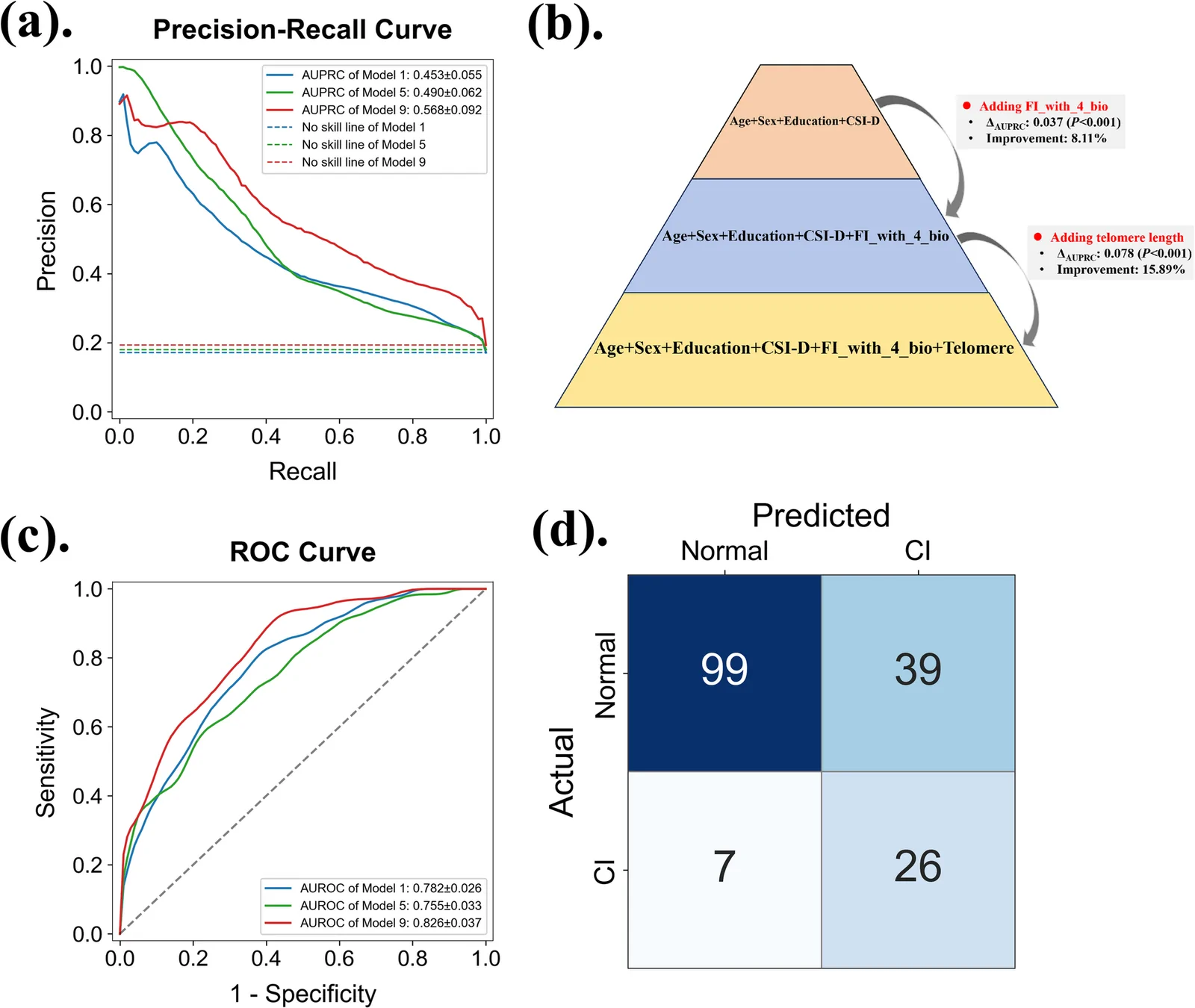
The prediction performance of penalized logistic regression models. **a** The precision-recall curves. **b** The change in AUPRC between models. c The receiver operating characteristic curves. **d** The confusion matrix of Model 9. AUPRC, AUROC, and confusion matrix were obtained through a 1000-time bootstrapping method

Figure 3 illustrates the comparative performance of models by progressively including BA indicators. The PR curves (Fig. 3-a) demonstrate progressive improvements from Model 1 (AUPRC: 0.453 ± 0.055) to Model 5 (AUPRC: 0.490 ± 0.062) and Model 9 (AUPRC: 0.568 ± 0.092), aligning with the changes in AUPRC in Fig. 3-b, where the improvements were 8.11% (adding FI with four biochemical markers) and 15.89% (further adding telomere length). ROC curves (Fig. 3-c) showed the sensitivity-specificity trade-offs across models, where Model 9 had the highest AUROC (0.826 ± 0.037). As an illustration, the confusion matrix of Model 9 was plotted in Fig. 3-d.

## Discussion

Our study demonstrates that a combination of conventional frailty index (without biochemical markers) with aging-related biochemical markers alongside telomere length provide moderate incremental value in predicting cognitive impairment among community-dwelling older adults, beyond conventional cognitive tests and general frailty measures (frailty phenotype and frailty index without biochemical markers). The findings may offer a multidimensional approach to assessing biological aging that connects to cognitive outcomes in older people.

Our results align with and extend current understanding of BA and its relationship with CI. First, the predictive utility of our biochemical marker-enriched FI supports existing evidence linking multi-system biological pathways to cognitive decline, including chronic inflammation (like hsCRP) [33], metabolic dysregulation (like creatinine and homocysteine) [34, 35], and micronutrient deficiency (like 25(OH)-D) [36]. This suggests that concurrent assessment of inflammatory, metabolic, and nutritional pathways may provide a more comprehensive quantification of aging burden than any single system alone. This supports emerging concepts of “inflammaging” and metabolic aging as drivers of neurodegeneration [37, 38]. Further, the incremental value of telomere length corroborates recent studies demonstrating its association with accelerated biological aging [39]. Our findings demonstrated that the added value of telomere length differed across frailty-related models, with its effect size of AUPRC change corresponding to small (for frailty phenotype), small (for FI with two biomarkers), and medium-to-large (for FI with four biomarkers). Notably, telomere length is influenced by a multitude of factors beyond aging, including genetics, chronic psychosocial stress, lifestyle, and environmental exposures [40, 41]. These factors contribute to the inter-individual variability in telomere length and its association with health outcomes. While these influencing factors for telomere length was not assessed in the current analysis, as the primary aim was to evaluate the incremental predictive value of telomere length within a biological aging metric for cognitive impairment. Future studies incorporating detailed assessments of these factors would be valuable to disentangle the specific drivers of telomere attrition in the context of cognitive aging.

Several additional findings also warrant further attention. First, we observed that the baseline CSI-D score demonstrated stronger predictive power for cognitive impairment compared to MMSE score. This likely reflects the more comprehensive assessment capacity of CSI-D test, which combines both participant cognitive testing and informant-reported functional decline, thereby capturing a broader spectrum of dementia-related impairments [24]. In contrast, the MMSE mainly focuses on global cognition and its ceiling effects in highly educated individuals may limit its sensitivity for detecting early or subtle cognitive decline [42]. While it's also worth noting that we incorporated baseline cognitive scores and educational attainment in the reference model. This approach was based on the following considerations: First, although individuals with CI at baseline were excluded in this study, inter-individual differences in cognitive performance may still exist among cognitively normal adults. Therefore, baseline cognitive scores were included to account for their influence on future CI risk. Second, while the inclusion of education may amplify its prediction ability for CI risk stratification, it was considered as a conservative measure to enable a more rigorous assessment of the incremental predictive value of BA indicators, by ensuring that their predictive gains are independent of educational influence. Consequently, the primary focus of our interpretation is not on the absolute effect of education itself, but on the added value of BA predictors. In support of this, additional analyses also confirmed that the conlusions remained unchanged even when education was excluded from the models (results not shown). Second, our findings also revealed that the added values of traditional biological aging measures including frailty phenotype and FI without biochemical markers were not statistically significant for cognitive impairment prediction compared to cognitive test scores. This may stem from the fact that conventional frailty primarily captures macroscopic manifestations of aging, whereas molecular-level changes incorporated in our model likely reflect earlier, more subtle pathological processes preceding overt cognitive symptoms [43]. We also found that the FI with two biochemical markers showed no added value for predicting CI compared to baseline cognitive score, this may suggest that a critical threshold of multisystem biochemical indicators is required to meaningfully augment cognitive risk prediction [44]. Isolated or limited markers may fail to capture the complex, interconnected nature of biological aging processes driving cognitive decline. Another possible explanation may be that when constructing the frailty index, the effect of the limited number of biomarkers may have been diluted by the abundance of clinical deficit indicators. Naturally, our findings may also be constrained by the limited sample size, necessitating further validation in larger populations in future. Finally, we noticed that only creatinine but not the other three biochemical markers and telomere length was associated with CI in descriptive analysis. Despite this, when combined into a frailty index, the four biochemical markers together demonstrated added value for predicting incident CI, with a small-to-medium effect size. Especially when further adding telomere length, the effect size of AUPRC change was medium-to-high. This finding may attribute to the following reasons: (1) Creatinine serves as an integrative biomarker, while traditionally viewed as a renal function marker, creatinine in older adults predominantly reflects muscle mass due to its generation from creatine phosphate in skeletal muscle. Diminished creatinine levels likely indicate sarcopenia, the age-related loss of muscle mass and function, which has been independently linked to cognitive decline [45]. Our studies also showed that incident CI groups had much lower baseline creatinine level, which confirmed the above explanation. Furthermore, low creatinine may also signal underlying malnutrition, particularly protein-energy wasting, which deprives the brain of essential nutrients and contributes to neurodegenerative processes [46]. In this predictive modelling study, we did not assess the influence of malnutrition on the predictive power of the BA indicator. Future studies could employ the GLIM model [47] to account for the effect of malnutrition on cognitive aging. (2) The relatively small sample size in our study may have limited power to detect subtle group differences. (3) The above discrepancy may also reflect the inherent differences between prediction and association analyses, with the former focusing more on identifying predictors rather than quantifying effect sizes. Lots of studies have pointed out that predictive importance does not guarantee biological significance, which means a strong predictive factor may not be statistically associated with outcome, likewise, variables with large effect sizes and low *P* values can also offer poor discriminative utility [48, 49]. Nevertheless, the penalized modelling approach holds a distinct advantage over conventional regression methods in small-sample scenarios, potentially uncovering subtle yet meaningful predictive gains that traditional models might miss.

### Strengths, implications, and limitations

Our study had some strengths and clinical implications. We integrated biochemical markers into the frailty index, demonstrating that a multidimensional assessment of biological aging could enhance CI prediction beyond conventional cognitive tests and general frailty measures. Besides, the simultaneous comparison of multiple BA indicators within a single aging cohort provided a unified way to evaluate their predictive utility for estimating CI risk. Additionally, the use of penalized regression and bootstrap techniques could help us obtain more robust performance estimates. However, our study also had some limitations. First, the reliance on MMSE and CSI-D scales to define outcomes in this study may not capture the full spectrum of cognitive impairment, as MMSE is subject to ceiling effects [42]. Also, some participants lacked information on informant interviews in CSI-D test, which may also influence the accuracy of cognitive assessment. However, the concurrent use of both cognitive tests helped mitigate these limitations to some extent. Future studies incorporating more objective cognitive assessments, such as the MoCA test or clinical diagnoses, are needed to validate these findings. Second, only four aging-related biochemical markers were included in the analysis due to data constraints. Consequently, their individual effects may have been diluted by other clinical deficits when calculating the frailty index. This may explain why not all FI versions demonstrate consistent predictive values. Third, while the AUPRC was employed to evaluate model performance given the class imbalance in our data, we acknowledge its potential limitations. Specifically, AUPRC may introduce biases in certain screening contexts, especially for the algorithmic unfairness issue [50]. Although the primary objective of this study was to assess the incremental predictive value of biological aging indicators, it is crucial to note that future investigations should incorporate fairness considerations prior to practical deployment. Fourth, we acknowledge the existence of multidimensional biological age measures that integrate functional, biochemical, and molecular components [51]. While we were unable to account molecular components in our analysis due to data constraints, and future work can compare the utility of more BA measures. Finally, this study had a relatively high loss-to-follow-up rate, and comparative analysis revealed that those lost to follow-up were a bit older and had slightly higher frailty levels than the included population. This may have led to some underestimation of the predictive performance of BA. Nevertheless, we found that the differences in baseline characteristics between the included and excluded populations were generally small, with most showing only small or small-to-medium effect sizes. Future studies are needed to more precisely quantify the predictive value of BA indicators in larger populations.

## Conclusion

The combination of conventional frailty index (without biochemical markers) with aging-related biochemical markers alongside telomere length provided moderate incremental value in predicting incident cognitive impairment beyond conventional cognitive tests among older adults, highlighting the importance of multi-system biological aging assessment in risk stratification. However, the findings need to be further validated in large populations and compared with other emerging biological aging markers.

## Supplementary Information

Supplementary Material 1.

## Data Availability

The data that support the findings of this study are available from the corresponding author upon reasonable request.

## Abbreviations

BA: Biological aging
CI: Cognitive impairment
FI: Frailty index
CHS: Cardiovascular Health Study
BMI: Body mass index
PASE: Physical activity scale of the elderly
eGFR: Estimated glomerular filtration rate
MMSE: Mini-Mental State Examination
CSI-D: Community Screening Instrument for Dementia
AUPRC: Area under the precision-recall curve
AUROC: Area under the ROC curve
