# Correction: The incremental predictive value of biological aging indicators for cognitive impairment in older adults: a longitudinal analysis on the Mr. OS & Ms. OS cohort

**DOI:** 10.1186/s13195-026-02198-y

**Published:** 2026-09-29

**Authors:** Yafei Wu, Ting Zhang, Tung Wai Auyeung, Jenny Lee, Jason Leung, Timothy Kwok

**Affiliations:** 1 Department of Medicine and Therapeutics, The Chinese University of Hong Kong, Shatin, New Territories, Hong Kong SAR, China; 2 Department of Geriatrics, Renji Hospital, School of Medicine, Shanghai Jiaotong University, Shanghai, 200127 China; 3 Jockey Club Institute of Ageing, The Chinese University of Hong Kong, Shatin, New Territories, Hong Kong SAR, China; 4 Jockey Club Centre for Osteoporosis Care and Control, The Chinese University of Hong Kong, Shatin, New Territories, Hong Kong SAR, China

**Correction: Alz Res Therapy 18, 9 (2026)**

**https://doi.org/10.1186/s13195-025-01917-1**

Following publication of the original article [1], some minor errors were noticed. The details of the corrections are as follows:

1. In the Methods section, under “Assessment of cognitive function” (Main text):
  Incorrect text: “dfscore ≥ 1.20 or cogscore ≥ 28.4”. Corrected text: “dfscore ≥ 0.120 or cogscore ≤ 29.5”.
    Additionally, the reference (reference #25) cited in this sentence should be replaced with: “Chan TS, et al. Validity and applicability of the Chinese version of community screening instrument for dementia. Dement Geriatr Cogn Disord. 2003;15(1):10-8”.
2. In the Results section, under “Incremental value of BA indicators in predicting CI” (Main text):
  Incorrect text: “0.112 (small) for the two-biomarker FI model (Model 7 vs. Model 3), and 0.706 (medium-to-high) for the four-biomarker FI model (Model 8 vs. Model 4)”.
    Corrected text: “0.112 (small) for the two-biomarker FI model (Model 8 vs. Model 4), and 0.706 (medium-to-high) for the four-biomarker FI model (Model 9 vs. Model 5)”.
3. In the Discussion section, under the third paragraph (Main text):
  Incorrect text: “Despite observing a trend towards worse conditions in the other three biochemical markers and telomere length for the CI group, the relatively small sample size in our study may have limited power to detect subtle group differences”. Corrected text: “The relatively small sample size in our study may have limited power to detect subtle group differences”.
4. In Text S1 (supplementary file):
  Incorrect text: “In our study, as the missing rate for every variable at the individual level was below this 20% threshold”.
    Corrected text: “In our study, as the missing rate at individual level was below the 20% threshold”.
5. In Table S3 (supplementary file):
  “MMSE (median, IQR)” should be “MMSE (mean ± std)”. “CSI-D (median, IQR)” should be “CSI-D (mean ± std)”. Accordingly, the first sentence in this table notes should be changed from “Continuous variables were expressed as mean (std) or median (IQR), and were compared by t-test or Wilcoxon rank sum test where appropriate” to “Continuous variables were expressed as mean (std), and were compared by t-test”.

The original article [1] and supplementary file have been corrected.

## Supplementary Information

Supplementary Material 1.
