# Supplementary material for: The incremental predictive value of biological aging indicators for cognitive impairment in older adults: a longitudinal analysis on the Mr. OS & Ms. OS cohort
Source: Alzheimers Res Ther. 2025 Dec 6;18:9. doi: 10.1186/s13195-025-01917-1 (PMC12797906; doi:10.1186/s13195-025-01917-1)
Supplement: Supplementary file 1 — Supplementary Material 1. [file 13195_2025_1917_MOESM1_ESM.docx]

**The incremental predictive value of biological aging indicators for cognitive impairment in older adults: Longitudinal analysis on the Mr. OS & Ms. OS cohort**

**Text S1.** Details about the construction of three frailty index versions

When constructing frailty index, we followed the recommendations by Kenneth Rockwood [1]. Specifically, the criteria for selecting FI items were as follows: 1) The prevalence must increase with age; 2) It should be associated with an adverse health outcome; 3) Not be too common before 65 years old (early saturation: prevalence>80%); 4) The prevalence should not be lower than 1%; 5) Represent multiple domains of functioning or multiple organ systems; 6) A missing proportion of <5% for each variable is recommended, and this threshold may be slightly adjusted based on research needs. In our study, we used 8% as the threshold (criteria 6) to ensure adequate sample size for constructing the biochemical marker-based frailty index.

In our analysis, only 7 variables contained missing data, namely hypersensitive C-reactive protein (hsCRP), 25-Hydroxyvitamin vitamin D [25(OH)-D], homocysteine, eGFR, 20cm narrow walk test, 5-time repeated standing test, and systolic blood pressure, with their missing proportions being 30.5%, 30.4%, 7.3%, 7.3%, 1.9%, 0.2%, and 0.1%. Therefore, the sample size was not influenced when calculating the conventional FI (without biochemical markers, *n*=1674) and the FI with two biochemical markers (including homocysteine and eGFR, *n*=1674), because all variables had a missing of being less than 8%. However, the high proportion of missing data (approximately 30%) for the two additional biomarkers (hsCRP and 25(OH)-D) precluded the construction of a reliable FI. Consequently, to adhere to the above FI construction criteria and maximize the analytical sample size, we excluded individuals with missing data for both hsCRP and 25(OH)-D. This approach resulted in missing data proportions of 7.2% for eGFR, 7.2% for homocysteine, 0.26% for hsCRP, and 0.09% for 25(OH)-D. These missing levels were deemed acceptable for constructing a four-biomarker-based FI (*n*=1166).

During the calculation of the individual FI, some variables may have missing data. In such cases, the denominator was adjusted by subtracting the number of missing deficits [2]. It is recommended that an FI score should not be calculated if more than 20% of the items are missing for an individual [3]. In our study, as the missing rate for every variable at the individual level was below this 20% threshold, therefore, FI scores were computed for all included participants.

Besides, in the baseline survey of Mr. OS and Ms. OS (Hong Kong) cohort, 826 participants had data on telomere length. Thus, with the inclusion of telomere length in the prediction models, the sample size was reduced across different scenarios, ranging from 570 (including both FI with four biochemical markers and telomere length) to 826 (including telomere and frailty phenotype or frailty index without biochemical markers or FI with two biochemical markers).

**Table S1**. The baseline characteristics of included and excluded participants

| Characteristics | Included (*n*=1674) | Died (*n*=352) | Lost to FU (*n*=1313) | Effect size ^a^ | Effect size ^b^ |
| --- | --- | --- | --- | --- | --- |
| Age | 70.7±4.2 | 74.7±5.4 | 73.1±5.1 | 0.087 | 0.577 |
| Sex (female) | 738 (44.1%) | 88 (25.0%) | 688 (52.4%) | 0.161 | 0.025 |
| Education |  |  |  | 0.103 | 0.120 |
| Illiterate | 244 (14.6%) | 48 (13.6%) | 321 (24.4%) |  |  |
| Primary or below | 811 (48.4%) | 200 (56.8%) | 632 (48.1%) |  |  |
| Secondary or above | 619 (37.0%) | 104 (29.5%) | 360 (27.4%) |  |  |
| MMSE score | 27.1±2.5 | 26.6±2.6 | 26.1±2.8 | 0.027 | 0.311 |
| CSI-D score | 30.9±1.3 | 30.6±1.5 | 30.4±1.6 | 0.025 | 0.312 |
| Frailty phenotype |  |  |  |  |  |
| Fit | 1093 (65.3%) | 148 (42.0%) | 648 (49.4%) | 0.139 | 0.189 |
| Pre-frail | 550 (32.9%) | 175 (49.7%) | 593 (45.2%) |  |  |
| Frail | 31 (1.9%) | 29 (8.2%) | 72 (5.5%) |  |  |
| FI_no_bio | 0.164±0.088 | 0.212±0.094 | 0.197±0.095 | 0.041 | 0.404 |
| FI_with_2_bio | 0.169±0.085 | 0.216±0.093 | 0.202±0.095 | 0.040 | 0.397 |
| FI_with_4_bio | 0.172±0.082 | 0.216±0.088 | 0.205±0.092 | 0.041 | 0.407 |
| Telomere length | 9.11±1.88 | 8.92±1.79 | 9.09±1.99 | 0.001 | 0.031 |

*Note*: mean (SD) and number (%) were used to describe continuous and categorial variables, respectively. FU, follow up. FI, frailty index. ^a^ Effect size was estimated for the three groups, and quantified by η² for continuous variables and Cramer’s V for categorical variables. ^b^ Effect size was estimated for two groups (died and lost to follow up groups were combined), and quantified by Cohen’s *d* for continuous variables and φ or Cramer’s V coefficients for categorical variables. η² with small, medium, and large effect sizes being defined by taking values of 0.01, 0.06, and 0.14, respectively. Cramer’s V or φ with small, medium, and large effect sizes being defined by taking values of 0.10, 0.30, and 0.50, respectively. Cohen’s *d* with small, medium, and large effect sizes being defined by taking values of 0.20, 0.50, and 0.80, respectively. We found that all differences of the characteristics except age between the included and excluded participants were small or small-to-medium effect level.

**Table S2**. Scoring methods of variables for constructing frailty index.

| **Variable** | **Scoring** |
| --- | --- |
| Diabetes | Yes (1 point) |
| Thyroid (low or high) | Yes (1 point) |
| Osteoporosis | Yes (1 point) |
| Stroke | Yes (1 point) |
| Hypertension | Yes (1 point) |
| Ischemic heart disease | Yes (1 point) |
| Congestive heart failure | Yes (1 point) |
| COPD | Yes (1 point) |
| Glaucoma | Yes (1 point) |
| Cataract | Yes (1 point) |
| Arthritis or gout | Yes (1 point) |
| Cancer | Yes (1 point) |
| Dizziness problem | Yes (1 point) |
| Fall | Yes (1 point) |
| Self-rated health [4] | 0, 0.25, 0.5, 0.75, 1 |
| Cannot walk 2-3 blocks | Have difficulty/Cannot do it (1 point) |
| Cannot climb 10 steps | Have difficulty/Cannot do it (1 point) |
| Cannot prepare meals | Have difficulty/Cannot do it (1 point) |
| Cannot do heavy housework | Have difficulty/Cannot do it (1 point) |
| Cannot do shopping | Have difficulty/Cannot do it (1 point) |
| Fracture | Yes (1 point) |
| Back pain | Yes (1 point) |
| Clumsy walking | Yes (1 point) |
| Clumsy using hands | Yes (1 point) |
| Medication [5] | Number of medications>=5 (Polypharmacy, 1 point) |
| Geriatric depression scale [6] | >=8 (1 point) |
| BMI [7, 8] | <18.5 kg/m2 (1 point) |
| ABI [9] | <0.9 (1 point) |
| Grip strength [10] | <28 kg for men and <18 kg for women (1 point) |
| Repeated chair stand [11] | Cannot finish, finishing with hands, finishing time>=12 seconds (1 point) |
| Six-meter walking time [11] | >=6 seconds (1 point) |
| 20cm Narrow Walk [12] | <0.78m/s (1 point) |
| SBP | SBP>140 or SBP<90 (1 point) |
| DBP | DBP>90 or DBP<60 (1 point) |
| Pulse [13] | Pulse<50 or Pulse>90 (1 point) |
| eGFR (Creatinine) [14] | eGFR<60 (1 point) |
| Homocysteine [15] | >13 μmol/L (1 point) |
| hsCRP [16, 17] | > 3 mg/L (1 point) |
| 25(OH)-D [18, 19] | <50 nmol/L (deficiency, 1 point) |

*Note*: COPD, chronic obstructive pulmonary disease; BMI, body fat mass; ABI, Ankle-Brachial Index; SBP, systolic blood pressure; DBP, diastolic blood pressure; eGFR, estimated Glomerular Filtration Rate; hsCRP, hypersensitive C-reactive protein; 25(OH)-D, 25-Hydroxyvitamin vitamin D. Number of medications were summarized based on the following types: Alpha-blocker, Androgen, Angiotensin converting enzyme (ACE) Inhibitor, Angiotensin II receptor antagonist, Antiandrogen, Antispasmodic, urinary, Aspirin, Benzodiazepine, Beta-blocker, Calcium channel blocker, Cholinergic agonist, Corticosteroid, inhaled, Corticosteroid, oral, Cox-II Inhibitor, Diuretic, loop, Diuretic, potassium-sparing, Diuretic, thiazide, Gemfibrozil, Histamine (H2) receptor antagonist, HMG CoA reductase inhibitor (statin), Hypoglycemic agents, Narcotic analgesic, Nitrate, Nonbenzodiazepine anticonvulsant, Nonsteroidal anti-inflammatory Agent (NSAID), Proton pump inhibitor, Selective serotonin reuptake inhibitor (SSRI), Sildenafil, Thyroid hormone, Trazodone, Tricyclic antidepressant. The estimated glomerular filtration rate (eGFR) was calculated by the 2021 CKD-EPI eGFR equation using serum creatinine, age, and sex.

**Table S3**. Comparisons of baseline predictors between training and test sets.

| Variables | Train set (70%) | Test set (30%) | *P*-value |
| --- | --- | --- | --- |
| Age (year, mean±std) | 70.7±4.2 | 70.8±4.3 | 0.599 |
| Female (*n*, %) | 507 (43.3%) | 231 (45.9%) | 0.321 |
| Education |  |  | 0.239 |
| Illiterate | 172 (14.7%) | 72 (14.3%) |  |
| Primary or below | 581 (49.6%) | 230 (45.7%) |  |
| Secondary or above | 418 (35.7%) | 201 (40.0%) |  |
| MMSE (median, IQR) | 27.1±2.5 | 27.0±2.5 | 0.817 |
| CSI-D (median, IQR) | 30.9±1.3 | 30.9±1.3 | 0.951 |
| Fried phenotype (*n*, %) |  |  | 0.436 |
| Fit | 771 (65.8%) | 322 (64.0%) |  |
| Pre-frail | 376 (32.1%) | 174 (34.6%) |  |
| Frail | 24 (2.0%) | 7 (1.4%) |  |
| FI_no_bio (mean±std) | 0.164±0.089 | 0.163±0.086 | 0.946 |
| FI_with_2_bio (mean±std) | 0.169±0.086 | 0.170±0.084 | 0.886 |
| FI_with_4_bio (mean±std) | 0.171±0.081 | 0.175±0.085 | 0.437 |
| Telomere length (mean±std) | 9.081±1.892 | 9.175±1.845 | 0.503 |
| Cognitive impairment (yes, *n*/%) | 199 (17.0%) | 86 (17.1%) | 0.959 |

*Note*: Continuous variables were expressed as mean (std) or median (IQR), and were compared by t-test or Wilcoxon rank sum test where appropriate. Categorical variables were expressed as number (percent), and were compared by chi-square test. The analytical sample size was 1674 for age, sex, education, MMSE, CSI-D, frailty phenotype, FI_no_bio, FI_with_2_bio, and cognitive outcome, 1166 for FI_with_4_bio, and 826 for telomere length.

**Table S4**. The prediction performance of models based on each biological aging indicators and cognitive tests alone on training and test sets

| Model | AUPRC | AUROC | BACC | SEN | PPV | F1 |
| --- | --- | --- | --- | --- | --- | --- |
| Test set | | | | | | |
| M1: MMSE | 0.332±0.049 | 0.686±0.033 | 0.612±0.027 | 0.373±0.052 | 0.342±0.047 | 0.356±0.045 |
| M2: CSID | 0.380±0.049 | 0.767±0.025 | 0.686±0.029 | 0.582±0.055 | 0.365±0.039 | 0.447±0.041 |
| M3: Phenotype | 0.169±0.036 | 0.492±0.029 | 0.500±0.000 | 1.000±0.000 | 0.171±0.016 | 0.292±0.023 |
| M4: FI_no_bio | 0.233±0.034 | 0.570±0.033 | 0.554±0.028 | 0.640±0.051 | 0.199±0.023 | 0.303±0.030 |
| M5: FI_with_2_bio | 0.230±0.034 | 0.558±0.034 | 0.559±0.027 | 0.348±0.049 | 0.239±0.037 | 0.282±0.039 |
| M6: FI_with_4_bio | 0.304±0.054 | 0.624±0.042 | 0.580±0.035 | 0.540±0.065 | 0.238±0.036 | 0.329±0.043 |
| M7: Telomere | 0.210±0.047 | 0.503±0.050 | 0.502±0.002 | 1.000±0.000 | 0.179±0.025 | 0.302±0.035 |
| Training set | | | | | | |
| M1: MMSE | 0.408±0.036 | 0.713±0.020 | 0.636±0.018 | 0.397±0.034 | 0.395±0.035 | 0.396±0.031 |
| M2: CSID | 0.411±0.035 | 0.723±0.020 | 0.654±0.019 | 0.512±0.036 | 0.341±0.028 | 0.409±0.028 |
| M3: Phenotype | 0.274±0.034 | 0.554±0.020 | 0.500±0.000 | 1.000±0.000 | 0.170±0.011 | 0.291±0.016 |
| M4: FI_no_bio | 0.256±0.026 | 0.604±0.023 | 0.563±0.019 | 0.676±0.034 | 0.201±0.015 | 0.310±0.021 |
| M5: FI_with_2_bio | 0.253±0.026 | 0.599±0.023 | 0.573±0.018 | 0.345±0.034 | 0.263±0.026 | 0.298±0.027 |
| M6: FI_with_4_bio | 0.261±0.032 | 0.579±0.028 | 0.571±0.023 | 0.560±0.043 | 0.229±0.023 | 0.324±0.028 |
| M7: Telomere | 0.221±0.030 | 0.541±0.031 | 0.502±0.002 | 1.000±0.000 | 0.180±0.015 | 0.305±0.022 |

*Note*: AUPRC, area under the precision-recall curve; AUROC, area under the receiver operating characteristic curve; BACC, balanced accuracy; SEN, sensitivity; PPV, positive predictive value; F1, F1 score. Phenotype, Fried phenotype; FI_no_bio, frailty index without biochemical markers; FI_with_2_bio, frailty index with eGFR and homocysteine included; FI_with_4_bio, frailty index with hsCRP and 25-OH Vitamin D further included. Telomere, telomere length. The results in the above table were expressed as mean and standard deviation by a 1000-time bootstrapping method.

**Table S5.** Comparisons of area under the precision-recall curve (AUPRC) between models based on each biological aging indicators and cognitive tests alone

| Model | Mean difference (95%CI) | Improvement | *P*-value | Cohen’s *d* |
| --- | --- | --- | --- | --- |
| M2-M1 | 0.048 (0.046-0.051) | 12.74% | <0.001 | 1.220 |
| M2-M3 | 0.211 (0.208-0.215) | 55.63% | <0.001 | 3.825 |
| M2-M4 | 0.147 (0.144-0.150) | 38.73% | <0.001 | 2.868 |
| M2-M5 | 0.150 (0.147-0.153) | 39.53% | <0.001 | 2.864 |
| M2-M6 | 0.076 (0.072-0.081) | 20.04% | <0.001 | 1.013 |
| M2-M7 | 0.170 (0.165-0.174) | 44.63% | <0.001 | 2.441 |
| M4-M5 | 0.003 (0.003-0.003) | 1.30% | <0.001 | 0.628 |
| M4-M6 | 0.071 (0.067-0.075) | 30.51% | <0.001 | 1.133 |

*Note*: The results were obtained by a 1000-time bootstrapping method. All differences are calculated by subtracting the first model's value from the second model's value. Cohen’s d was used to assess the effect size of change in AUPRC between models, and was categorized as small (d=0.2), medium (d=0.5), or large (d≥0.8). Models 1-7 were constructed by individually including one of the following factors: MMSE score, CSI-D score, frailty phenotype, frailty index without biomarker, frailty index with two biomarkers, frailty index with four biomarkers, and telomere length.

**Table S6.** The hyperparameters of prediction models that include each biological aging indicators and cognitive test alone

| Model | Optimal hyperparameters |
| --- | --- |
| M1: MMSE | C=0.01, penalty='l2', class_weight='balanced' |
| M2: CSID | C=10, penalty='l1', class_weight=[0: 1, 1: 4] |
| M3: Phenotype | C=0.001, penalty='l2', class_weight=[0: 1, 1: 6] |
| M4: FI_no_bio | C=0.01, penalty='l2', class_weight='balanced' |
| M5: FI_with_2_bio | C=1, penalty='l1', class_weight=[0: 1, 1: 4] |
| M6: FI_with_4_bio | C=1, penalty='l2', class_weight='balanced' |
| M7: Telomere | C=0.001, penalty='l2', class_weight='balanced' |

*Note*: ‘C’ is the inverse of regularization strength, smaller values indicate stronger regularization; ‘Penalty’ is the norm used in the penalization ('l1', 'l2'); ‘Class weight’ means weights associated with classes to handle imbalanced data (e.g., 'balanced', or the custom weights in this study. The weights before the square brackets refer to cognitively normal individuals (fixed at 1), and the weight after refers to cognitively impaired individuals.).

**Table S7**. The prediction performance of models by progressively including biological aging indicators (training set)

| Model | AUPRC | AUROC | BACC | SEN | PPV | F1 |
| --- | --- | --- | --- | --- | --- | --- |
| M1: Ref | 0.471±0.037 | 0.784±0.018 | 0.693±0.019 | 0.592±0.034 | 0.370±0.027 | 0.455±0.027 |
| M2: Ref+Phenotype | 0.479±0.037 | 0.784±0.018 | 0.686±0.019 | 0.512±0.037 | 0.429±0.032 | 0.466±0.030 |
| M3: Ref+FI_no_bio | 0.475±0.037 | 0.786±0.018 | 0.714±0.018 | 0.688±0.033 | 0.353±0.024 | 0.466±0.025 |
| M4: Ref+FI_with_2_bio | 0.474±0.037 | 0.786±0.018 | 0.703±0.019 | 0.602±0.035 | 0.387±0.028 | 0.471±0.028 |
| M5: Ref+FI_with_4_bio | 0.455±0.044 | 0.793±0.020 | 0.708±0.022 | 0.575±0.042 | 0.445±0.038 | 0.501±0.035 |
| M6: Ref+Phenotype+Telomere | 0.490±0.047 | 0.791±0.022 | 0.729±0.022 | 0.800±0.039 | 0.338±0.029 | 0.475±0.032 |
| M7: Ref+FI_no_bio+Telomere | 0.503±0.047 | 0.796±0.022 | 0.728±0.024 | 0.675±0.045 | 0.404±0.036 | 0.504±0.035 |
| M8: Ref+FI_with_2_bio+Telomere | 0.502±0.047 | 0.799±0.022 | 0.729±0.024 | 0.714±0.044 | 0.379±0.033 | 0.494±0.034 |
| M9: Ref+FI_with_4_bio+Telomere | 0.448±0.055 | 0.769±0.026 | 0.726±0.027 | 0.729±0.049 | 0.389±0.040 | 0.506±0.040 |

*Note*: Ref, reference (the reference model included age, sex, education, and baseline CSI-D score); AUPRC, area under the precision-recall curve; AUROC, area under the receiver operating characteristic curve; BACC, balanced accuracy; SEN, sensitivity; PPV, positive predictive value; F1, F1 score. Phenotype, Fried phenotype; FI_no_bio, frailty index without biochemical markers; FI_with_2_bio, frailty index with eGFR and homocysteine included; FI_with_4_bio, frailty index with hsCRP and 25-OH Vitamin D further included. The results in the above table were expressed as mean and standard deviation by a 1000-time bootstrapping method.

**Table S8**. Comparisons of area under the precision-recall curve (AUPRC) between models by progressively including biological aging indicators (test set)

| Model | Mean difference (95%CI) | Improvement | *P*-value | Cohen’s *d* |
| --- | --- | --- | --- | --- |
| M1-M2 | 0.015 (0.014-0.015) | 3.27% | <0.001 | 1.510 |
| M1-M3 | 0.004 (0.004-0.005) | 0.91% | <0.001 | 0.432 |
| M1-M4 | 0.009 (0.008-0.009) | 1.89% | <0.001 | 0.986 |
| M5-M1 | 0.037 (0.032-0.042) | 8.11% | <0.001 | 0.451 |
| M6-M2 | 0.009 (0.003-0.015) | 2.08% | 0.002 | 0.100 |
| M7-M3 | 0.001 (-0.005, 0.007) | 0.25% | 0.704 | 0.012 |
| M8-M4 | 0.010 (0.005-0.016) | 2.32% | <0.001 | 0.112 |
| M9-M5 | 0.078 (0.071-0.085) | 15.89% | <0.001 | 0.706 |

*Note*: All differences are calculated by subtracting the first model's value from the second model's value. *P*-values were calculated by t-test. Cohen’s *d* was used to quantify the effect size of change in AUPRC, and was categorized as small (d=0.2), medium (d=0.5), or large (d≥0.8). Model 1 includes age, sex, education, and baseline CSI-D score (reference model for comparison), then incorporating four individual frailty-related BA indicators (frailty phenotype, FI_no_bio, FI_with_2_bio, and FI_with_4_bio) into Model 1 (Models 2-5), further adding telomere length into Models 2-5 (Models 6-9).

**Table S9**. The optimal hyperparameters of models by progressively including biological aging indicators

| Model | Optimal hyperparameters |
| --- | --- |
| M1: Ref | C=1, penalty='l2', class_weight=[0:1,1:4] |
| M2: Ref+Phenotype | C=5, penalty='l1', class_weight=[0: 1, 1: 3] |
| M3: Ref+FI_no_bio | C=10, penalty='l2', class_weight='balanced' |
| M4: Ref+FI_with_2_bio | C=5, penalty='l2', class_weight=[0: 1, 1: 4] |
| M5: Ref+FI_with_4_bio | C=1, penalty='l1', class_weight=[0:1,1:3] |
| M6: Ref+Phenotype+Telomere | C=1, penalty='l2', class_weight=[0: 1, 1: 6] |
| M7: Ref+FI_no_bio+Telomere | C=1, penalty='l2', class_weight=[0: 1, 1: 4] |
| M8: Ref+FI_with_2_bio+Telomere | C=10, penalty='l2', class_weight='balanced' |
| M9: Ref+FI_with_4_bio+Telomere | C=50, penalty='l1', class_weight=[0: 1, 1: 4] |

*Note*: ‘C’ is the inverse of regularization strength, smaller values indicate stronger regularization; ‘Penalty’ is the norm used in the penalization ('l1', 'l2'), ‘Class weight’ means weights associated with classes to handle imbalanced data (e.g., 'balanced', or the custom weights in this study. The weights before the square brackets refer to cognitively normal individuals (fixed at 1), and the weight after refers to cognitively impaired individuals.). Ref, reference (the reference model included age, sex, education, and baseline CSI-D score).

**Reference**

1. Rockwood, K., Conceptual Models of Frailty: Accumulation of Deficits. Can J Cardiol, 2016. **32**(9): p. 1046-50.

2. Stuck, A.K., et al., Ability of 3 Frailty Measures to Predict Short-Term Outcomes in Older Patients Admitted for Post-Acute Inpatient Rehabilitation. J Am Med Dir Assoc, 2022. **23**(5): p. 880-884.

3. Theou, O., et al., How to construct a frailty index from an existing dataset in 10 steps. Age Ageing, 2023. **52**(12).

4. Searle, S.D., et al., A standard procedure for creating a frailty index. BMC Geriatr, 2008. **8**: p. 24.

5. Masnoon, N., et al., What is polypharmacy? A systematic review of definitions. BMC Geriatr, 2017. **17**(1): p. 230.

6. Sun, W.J., et al., Depressive symptoms and suicide in 56,000 older Chinese: a Hong Kong cohort study. Soc Psychiatry Psychiatr Epidemiol, 2012. **47**(4): p. 505-14.

7. Lv, Y., et al., The obesity paradox is mostly driven by decreased noncardiovascular disease mortality in the oldest old in China: a 20-year prospective cohort study. Nat Aging, 2022. **2**(5): p. 389-396.

8. Kıskaç, M., et al., What is the Optimal Body Mass Index Range for Older Adults? Ann Geriatr Med Res, 2022. **26**(1): p. 49-57.

9. Espinola-Klein, C., et al., Different calculations of ankle-brachial index and their impact on cardiovascular risk prediction. Circulation, 2008. **118**(9): p. 961-7.

10. Chen, L.K., et al., Asian Working Group for Sarcopenia: 2019 Consensus Update on Sarcopenia Diagnosis and Treatment. J Am Med Dir Assoc, 2020. **21**(3): p. 300-307.e2.

11. Tiedemann, A., et al., The comparative ability of eight functional mobility tests for predicting falls in community-dwelling older people. Age Ageing, 2008. **37**(4): p. 430-5.

12. Gimmon, Y., et al., Application of the clinical version of the narrow path walking test to identify elderly fallers. Arch Gerontol Geriatr, 2016. **63**: p. 108-13.

13. Nanchen, D., Resting heart rate: what is normal? Heart, 2018. **104**(13): p. 1048-1049.

14. Stevens, L.A., et al., Evaluation of the Chronic Kidney Disease Epidemiology Collaboration equation for estimating the glomerular filtration rate in multiple ethnicities. Kidney Int, 2011. **79**(5): p. 555-62.

15. Varga, E.A., et al., Cardiology patient pages. Homocysteine and MTHFR mutations: relation to thrombosis and coronary artery disease. Circulation, 2005. **111**(19): p. e289-93.

16. Wu, L., et al., Elevated high-sensitivity C-reactive protein levels increase the risk of new-onset cardiac conduction disorders. Cardiovasc Diabetol, 2023. **22**(1): p. 268.

17. Pearson, T.A., et al., Markers of inflammation and cardiovascular disease: application to clinical and public health practice: A statement for healthcare professionals from the Centers for Disease Control and Prevention and the American Heart Association. Circulation, 2003. **107**(3): p. 499-511.

18. Chan, R., et al., Association between serum 25-hydroxyvitamin D and psychological health in older Chinese men in a cohort study. J Affect Disord, 2011. **130**(1-2): p. 251-9.

19. Chen, J., et al., Vitamin D status among the elderly Chinese population: a cross-sectional analysis of the 2010-2013 China national nutrition and health survey (CNNHS). Nutr J, 2017. **16**(1): p. 3.
